# Modelling the cost-effectiveness of introducing subsidised malaria rapid diagnostic tests in the private retail sector in sub-Saharan Africa

**DOI:** 10.1136/bmjgh-2019-002138

**Published:** 2020-05-20

**Authors:** David Bath, Catherine Goodman, Shunmay Yeung

**Affiliations:** 1 Department of Health Services Research and Policy, London School of Hygiene and Tropical Medicine, London, UK; 2 Department of Global Health and Development, London School of Hygiene and Tropical Medicine, London, UK; 3 Department of Clinical Research, Department of Global Health and Development, London School of Hygiene and Tropical Medicine, London, UK

**Keywords:** malaria, diagnostics and tools, health economics

## Abstract

**Background:**

Over the last 10 years, there has been a huge shift in malaria diagnosis in public health facilities, due to widespread deployment of rapid diagnostic tests (RDTs), which are accurate, quick and easy to use and inexpensive. There are calls for RDTs to be made available at-scale in the private retail sector where many people with suspected malaria seek care. Retail sector RDT use in sub-Saharan Africa (SSA) is limited to small-scale studies, and robust evidence on value-for-money is not yet available. We modelled the cost-effectiveness of introducing subsidised RDTs and supporting interventions in the SSA retail sector, in a context of a subsidy programme for first-line antimalarials.

**Methods:**

We developed a decision tree following febrile patients through presentation, diagnosis, treatment, disease progression and further care, to final health outcomes. We modelled results for three ‘treatment scenarios’, based on parameters from three small-scale studies in Nigeria (TS-N), Tanzania (TS-T) and Uganda (TS-U), under low and medium/high transmission (5% and 50% *Plasmodium falciparum* (parasite) positivity rates (PfPR), respectively).

**Results:**

Cost-effectiveness varied considerably between treatment scenarios. Cost per disability-adjusted life year averted at 5% PfPR was US$482 (TS-N) and US$115 (TS-T) and at 50% PfPR US$44 (TS-N) and US$45 (TS-T), from a health service perspective. TS-U was dominated in both transmission settings.

**Conclusion:**

The cost-effectiveness of subsidised RDTs is strongly influenced by treatment practices, for which further evidence is required from larger-scale operational settings. However, subsidised RDTs could promote increased use of first-line antimalarials in patients with malaria. RDTs may, therefore, be more cost-effective in higher transmission settings, where a greater proportion of patients have malaria and benefit from increased antimalarial use. This is contrary to previous public sector models, where RDTs were most cost-effective in lower transmission settings as they reduced unnecessary antimalarial use in patients without malaria.

**Key questions**

**What is already known?**

- Over the last 10 years, rapid diagnostic tests (RDTs) have played an important role in the substantial increase in parasitological confirmation of suspected malaria cases in primary level public health facilities.
- In many settings, private retailers, such as drug shops and small pharmacies, provide the majority of antimalarials but rarely carry out parasitological diagnosis.
- Evidence of the effectiveness of subsidised malaria RDTs in the private retail sector in sub-Saharan Africa (SSA) is limited to small-scale trials and pilot studies.

**What are the new findings?**

- RDT cost-effectiveness in the SSA private retail sector is strongly influenced by treatment practices, for which further evidence is required from larger-scale operational settings.
- Initial evidence indicates that subsidised RDTs may promote the increased use of antimalarials in patients with malaria.

**What do the new findings imply?**

- RDT introduction may be more cost-effective in higher transmission settings, where a greater proportion of febrile patients have malaria and therefore benefit from increased antimalarial use.
- These findings challenge the traditional view of RDTs as primarily a way to reduce inappropriate antimalarial use and improve case management of non-malaria cases.

## Introduction

It is now over a decade since WHO first recommended parasitological confirmation prior to treatment for all suspected malaria cases.^1^ Over this period, there has been a major increase in parasitological diagnosis in public health facilities, with policy implementation accelerated by two key developments. First, inexpensive but ineffective antimalarials, such as chloroquine, were replaced with much more expensive artemisinin combination therapies (ACTs), heightening concerns about the waste of medicines arising from presumptive treatment. This coincided with increased availability of malaria rapid diagnostic tests (RDTs), which are quick (<20 min), accurate, simple to use, relatively inexpensive and avoid the need for functioning microscopes and trained microscopists. In 2017, 82% of suspected public sector malaria cases in the WHO African Region received a malaria diagnostic test, compared with 36% in 2010^2 3^ and it is estimated that RDTs now account for three-quarters of all such tests conducted.^3^

There are increasing calls for RDTs to be made available at-scale in the private sector as well—in particular, the private retail sector where a high proportion of people with suspected malaria seek care.^4–6^ Private retailers, primarily drug shops and small pharmacies, provide the majority of antimalarials in many settings but rarely carry out parasitological diagnosis. In a study across eight countries in sub-Saharan Africa (SSA) (2014–2015), in five countries RDTs were available in less than 10% of outlets that stocked antimalarials, with the highest availability in Uganda being only 21.5%.^7^ Such retailers vary substantially in terms of the qualifications of staff, from qualified pharmacists to drug sellers with no formal health training, and the types of prescription drugs they are permitted to sell.^8 9^ A high proportion of patients sold antimalarials at retail outlets do not have malaria parasitaemia, while those with malaria often receive no antimalarial or a less effective antimalarial monotherapy instead of an ACT.^10^ It has been argued that the increased availability of RDTs in these settings would better target antimalarial treatment to malaria patients and improve case management of so-called ‘non-malarial febrile illness’ (NMFI).^11 12^ However, there are also concerns of misdiagnosis due to a lack of provider training and supervision, continued overtreatment of test negatives with ACTs, and unsafe handling of infectious waste.^8^

Since 2010, there have been moves to increase accessibility and affordability of quality-assured ACTs in the private sector, initially through the Global Fund’s Affordable Medicines Facility-malaria and subsequently the Private Sector Co-payment Mechanism, which subsidised ACT prices through a co-payment at the manufacturer level.^13 14^ It has been argued that a similar subsidy of RDTs, together with a continued ACT subsidy, could promote private sector RDT uptake and incentivise patients to purchase an RDT instead of presumptive treatment.^15^

Robust evidence on the impact and value-for-money of retail sector RDT introduction is not yet available. While subsidised RDTs have been provided through the retail sector in parts of Asia for more than 15 years,^16^ in SSA they are still mainly limited to small-scale trials and pilot studies.^8 17^ There is considerable variation across these studies in the impact of RDT introduction on the likelihood of receiving ‘appropriate’ treatment (eg, an ACT for malaria or an antibiotic for a bacterial infection).^8^ There is only one published empirical economic evaluation of RDT introduction in the SSA retail sector,^18^ which reports cost-effectiveness in terms of intermediate outcomes (cost per appropriately treated patient)^18^ rather than final health outcomes (such as disability-adjusted life years (DALYs) averted).^19^

To our knowledge, this paper is the first to model the cost-effectiveness of subsidised malaria RDT introduction in the SSA private sector. Unlike a single empirical study, a modelling approach enables exploration of cost-effectiveness under a range of treatment practices, and varying levels of malaria transmission, as well as assessing sensitivity to the many other model parameters. We assess retail sector RDT introduction in the context of an existing ACT subsidy, as the empirical evidence is drawn from such settings. We apply our model to the context of *Plasmodium falciparum* malaria in SSA, which accounts for over 90% of annual malaria deaths globally.^3^ We build on previous models of RDT introduction in the public sector^20–22^ and incorporate a number of methodological enhancements including: (1) consideration of patients coinfected with malaria and bacteria; (2) relaxation of the assumption that all patients in the intervention arm receive an RDT; (3) inclusion of treatment with both ACT and non-ACT antimalarials, as well as combinations of different treatments; and (4) accounting for the impact of poor quality drugs and imperfect adherence to treatment by patients in estimating treatment effectiveness.

## Methods

### Intervention and model structure

We developed a decision-analytical model to estimate the incremental costs and outcomes of large-scale (eg, national) introduction of subsidised RDTs in the retail sector in a theoretical SSA setting (‘intervention’), against a comparator of no retail sector RDT introduction (‘control’). The control arm includes an 80% subsidy of ACTs in the private sector but no availability of RDTs in the retail sector. The intervention also includes an 80% ACT subsidy but with RDTs available at retail outlets, subsidised by 50% to improve affordability, and supporting interventions: community sensitisation, training of providers, disposal of waste, and ongoing provider monitoring (table 1).

**Table 1:** Description of intervention and control arms

**Control (no RDTs**) No RDTs available in private retail outlets. ACTs subsidised in the private sector.	**Intervention (RDTs available**) Introduction of RDTs, with 50% subsidy, in private retail outlets (uptake 41%). Supporting interventions: community sensitisation on the benefits and availability of RDTs, training of providers in safe and effective RDT use and case management protocols (3–4 day workshop), monitoring and supervision of outlets, waste disposal. ACTs subsidised in the private sector.

ACTs, artemisinin combination therapies; RDTs, rapid diagnostic tests.

**Figure 1:**
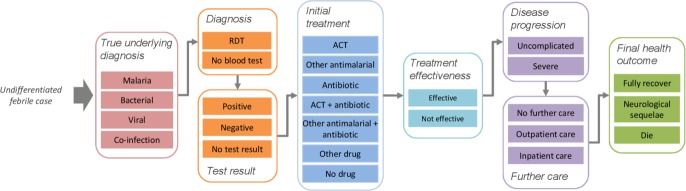
Key nodes and branches of decision-analytical model pathway of undifferentiated febrile case, from initial presentation at a retail outlet, through diagnosis and initial treatment, the effectiveness of any treatment, disease progression, further care, to final health outcomes. ACT, artemisinin combination therapy; RDT, rapid diagnostic test.

In line with previous public sector models,^20–22^ we use a decision tree that follows febrile patients from initial presentation at a retail outlet, through diagnosis and initial treatment, the effectiveness of any treatment, possible disease progression and further care, to their final health outcomes (figure 1, online supplementary file S1). Parameter values for RDT uptake, initial treatment, and supporting intervention costs were taken from selected empirical studies of subsidised RDT introduction in the retail sector. All other parameters draw on a wide range of secondary sources, including previous public sector models. Key model parameters are shown in table 2, and all other parameters in the online supplementary file S2. We conducted the analysis for a notional cohort of 100 000 patients with uncomplicated febrile illness without obvious cause, presenting at retail outlets, at two levels of malaria transmission: 5% *P*. *falciparum* positivity rate (PfPR) (a ‘low’ transmission setting) and 50% PfPR (a ‘medium/high’ transmission setting). We conducted sensitivity analysis to explore cost-effectiveness across the full range of PfPR (0%–90%). For parameters that vary depending on the intensity of malaria transmission (eg, case fatality rate for untreated malaria), we assumed different values for the ‘low’ and ‘medium/high’ transmission settings.^21 23^ Patients presenting with fever are classified according to their true underlying diagnosis as either: malaria, bacterial, malaria and bacterial coinfection, or viral only (online supplementary file S3).

10.1136/bmjgh-2019-002138.supp1 Supplementary data

**Table 2:** Key cost-effectiveness model parameters

Parameter	Best estimate (range for PSA)		Distribution for PSA	Source(s)
Proportion of patients under 5 years	0.40 (0.30–0.50)		Beta	20
**True underlying diagnosis**				
Malaria cases with bacterial coinfection	0.06 (0.03–0.174)		Beta	61 62
NMFI cases that are bacterial infection	0.10 (0.013–0.15)		Beta	20 63
**Diagnosis (intervention arm only)**				
Patients receiving RDT (‘uptake’)	0.41 (0.08–0.72)		Beta	8
RDT sensitivity	0.948 (0.931–0.961)		Beta	64
RDT specificity	0.952 (0.631–0.967)		Beta	29 64
**Initial treatment**				
Proportion of patients receiving an ACT, non-ACT antimalarial, antibiotic, other drug, or no drug	See figure 2, online supplementary file S4		Dirichlet	28 30 31 36–38
**Treatment effectiveness**				
ACT efficacy (for malaria)	0.955 (0.82–1.00)		Beta	39
Other antimalarial efficacy (for malaria)	0.78 (0.183–0.97)		Beta	39
Antibiotic efficacy (for bacterial infection)	0.80 (0.72–0.88)		Beta	Assumption (range: ±10%)
Proportion of stated API in drug (drug quality)	0.92 (0.828–1.011)		Gamma	40 (range: ±10%)
Proportion of required dose consumed (adherence to treatment)	0.892 (0.5–1.0)		Beta	41 (range: assumption)
Reduction in treatment efficacy due to API consumed	Low transmission:	Medium/high transmission:		Assumptions
80%–85%	0.15 (0.05–0.25)	0.10 (0.00–0.20)	Beta	
75%–80%	0.30 (0.15–0.45)	0.25 (0.10–0.40)	Beta	
50%–75%	0.60 (0.45–0.75)	0.50 (0.35–0.65)	Beta	
<50%	0.95 (0.90–1.00)	0.95 (0.90–1.00)	Beta	
**Disease progression and further care**				
Malaria case progresses to severe with no (or not effective) treatment	Low transmission:	Medium/high transmission:		65 (Medium/high transmission best estimates: assumptions)
<5 years	0.30 (0.10–0.90)	0.10 (0.05–0.60)	Beta	
5+ years	0.18 (0.05–0.50)	0.02 (0.00–0.15)	Beta	
Bacterial case progresses to severe with no (or not effective) treatment	Low HIV:	High HIV:		65
<5 years	0.20 (0.05–0.80)	0.40 (0.15–0.90)	Beta	
5+ years	0.20 (0.05–0.70)	0.30 (0.10–0.90)	Beta	
Severe case receives further (inpatient) care	0.75 (0.19–0.88)		Beta	Assumption (range^20^)
**Final health outcomes**				
CFR of severe malaria receiving inpatient care	0.10 (0.05–0.15)		Beta	20 42
CFR of severe malaria with no further care	Low transmission:	Medium/high transmission:		65
<5 years	0.73 (0.25–0.95)	0.45 (0.05–0.90)	Beta	
5+ years	0.70 (0.30–0.95)	0.60 (0.10–0.90)	Beta	
CFR of severe bacterial infection receiving inpatient care	0.15 (0.10–0.20)		Beta	20
CFR of severe bacterial infection with no further care	Low HIV:	High HIV:		
<5 years	0.40 (0.10–0.90)	0.50 (0.15–1.00)	Beta	65
5+ years	0.30 (0.10–0.80)	0.50 (0.10–0.90)	Beta	
Implementation costs (2017 US$)*				
RDT ex-manufacturer price	0.22 (0.17–0.28)		Gamma	66 (range: ±25%)
RDT subsidy (% ex-manufacturer price)	0.50 (0.40–0.60)		Beta	Assumption
ACT ex-manufacturer price	0.68 (0.51–1.56)		Gamma	67
ACT subsidy (% ex-manufacturer price)	0.80 (0.70–0.90)		Beta	Assumption
Inpatient cost per day†	4.33 (3.25–17.72)		Gamma	68
Supporting intervention cost per febrile patient	0.43 (0.21–0.64)		Gamma	See online supplementary file S5

*All costs were adjusted to 2017 US dollars using the median of the five year annual average GDP deflator in the six countries participating in the Private Sector Co-payment Mechanism.^69^

†Inpatient cost is bed-day cost only; excludes cost of treatment.

ACT, artemisinin combination therapy; API, active pharmaceutical ingredient; CFR, case fatality rate; GDP, gross domestic product; NMFI, non-malarial febrile illness; PSA, probabilistic sensitivity analysis; RDT, rapid diagnostic test.

**Figure 2:**
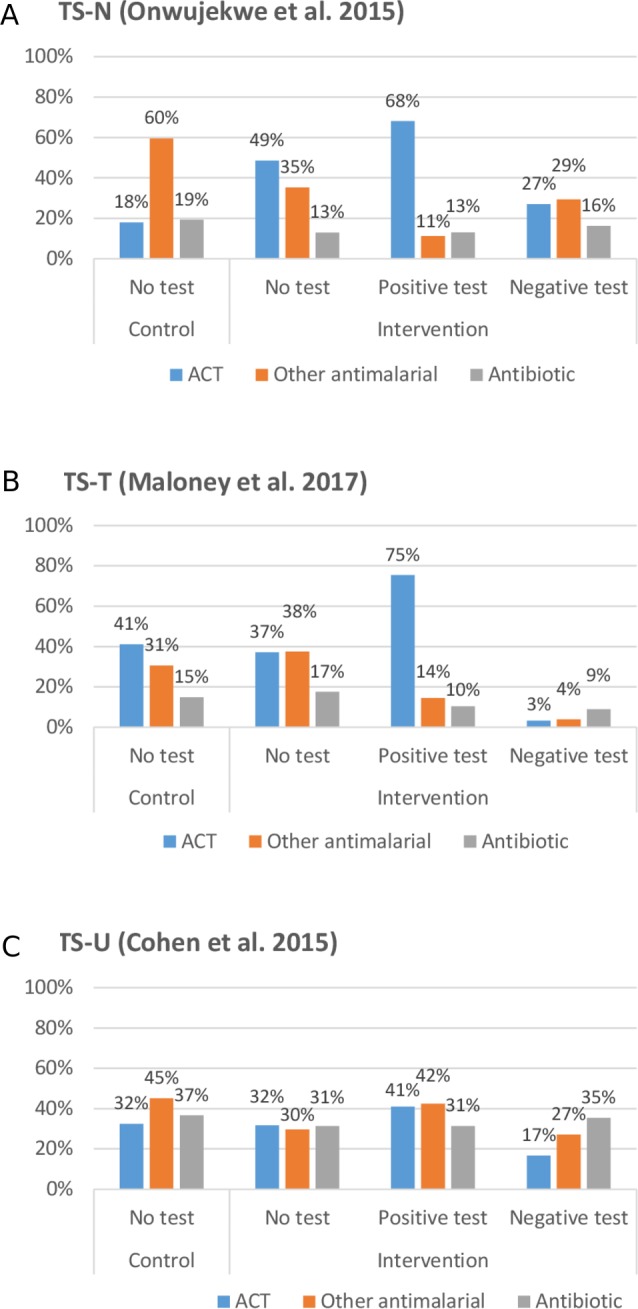
Comparison of initial treatment received in private retail outlets without RDT availability (control) and with RDT availability (intervention) by test result, across three treatment scenarios: TS-N, TS-T, TS-U. ACT, artemisinin combination therapy; RDT, rapid diagnostic test; TS-N, TS-T, TS-U, three small-scale studies in Nigeria, Tanzania and Uganda.

#### Diagnosis and initial treatment

Estimates of the proportion of patients in the intervention arm that receive an RDT (‘uptake’) were taken from a literature review by Visser *et al* of empirical studies of RDT introduction in the retail sector.^8^ We excluded one study conducted outside of SSA,^24^ two studies where RDTs were provided at no cost to patients,^25 26^ and one study where information on uptake was not available.^27^ We also excluded two further studies where data on RDT uptake were obtained from provider records rather than mystery shoppers or exit interviews,^28 29^ as providers may exaggerate their adherence to study protocols in their own records. RDT uptake in the intervention arm was estimated as the median uptake (41%) of the nine intervention arms across the six included studies.^30–35^ The remaining patients in the model’s intervention arm and all patients in the control arm did not receive a malaria diagnostic test prior to treatment. We also tested the sensitivity of results to changes in RDT uptake, based on the lowest (8%) and highest (72%) uptake from the included studies.

To obtain sources for initial treatment parameters, we identified published studies of RDT introduction in the SSA retail sector. We excluded studies where the RDT was provided for free,^25 26^ data on initial treatment were obtained from provider records^29^ or baseline and endline data were obtained from different types of data collection.^32^ This led to the inclusion of three eligible studies.^28 30 31^ As these three studies reported substantially different impacts of RDT introduction on the use of ACTs and other antimalarials, we modelled cost-effectiveness separately using initial treatment parameters from each of these three studies as three ‘treatment scenarios’. The three scenarios were based on: a cluster-randomised trial at private retailers (including pharmacies and drug shops) and public health facilities in two sites in southeastern Nigeria (TS-N)^31^; a non-randomised controlled trial in accredited drug dispensing outlets in two districts in Tanzania (TS-T)^30^; and a cluster-randomised trial of drug shops in 79 villages in eastern Uganda (TS-U).^28^ We liaised with authors to obtain additional data on all drugs received and, in the case of TS-N, to separate retail sector from public sector patients.^36–38^ Data were categorised into seven mutually exclusive treatment categories (see online supplementary file S4). Figure 2 shows the treatment received under each scenario, with and without RDT availability. The treatment scenarios differ considerably: for TS-N, RDT introduction increased ACT use (and reduced other antimalarial use) even for those not tested or with a negative test, whereas TS-T shows an increase in ACT use for test-positives and a reduction for test negatives. TS-U shows a similar but more modest impact on ACT use.

#### Treatment effectiveness

We estimated the success of initial treatment in curing the true underlying diagnosis in a ‘real world’ setting (‘treatment effectiveness’). We calculated the probability of treatment effectiveness for both antimalarials and antibiotics as the efficacy of each treatment less a percentage reduction based on the proportion of active pharmaceutical ingredient (API) consumed by a patient (table 2). Antimalarial efficacy estimates were based on the median day 28 success rate in SSA clinical trials reported by the Worldwide Antimalarial Resistance Network^39^; antibiotic efficacy was based on expert opinion. We estimated API consumed as the product of the average percent API per dose (which is less than 100% due to imperfect drug quality) and the average percent of a dose consumed (due to patient adherence to treatment), based on studies of antimalarial quality^40^ and patient adherence.^41^

#### Disease progression and further care

We assumed that viral infections are self-resolving. For infections due to malaria and/or bacterial infection, where initial treatment with antimalarials or antibiotics is effective we assumed that illness resolved without further care. Where no initial treatment for malaria or bacterial infection is received, we assumed that the disease may progress from an uncomplicated to a severe state, using estimates from a published Delphi survey of malaria experts.^23^ In the absence of data on outcomes of those receiving ineffective treatment, we assumed that the probability of progression was the same for untreated infections. We explore the uncertainty of disease progression, across a wide range of values, via sensitivity analysis.

#### Final health outcomes

Where illness remains uncomplicated, full recovery is assumed. Where illness has progressed to severe, different case fatality rates are assumed based on the true underlying diagnosis and whether further inpatient or outpatient care is provided.^20 23 42 43^ In addition to death, patients with severe febrile illness risk long-term neurological sequelae.^20 42 43^

### Costs

We estimated incremental costs as the difference between costs in the intervention and control arms, from both health service and societal perspectives, in 2017 US dollars. Health service costs included the subsidies on RDTs and ACTs, the cost of further outpatient or inpatient care at public health facilities, and supporting intervention costs. Societal costs comprised health service costs plus direct patient out-of-pocket medical costs for initial treatment (including the retail price of RDTs and drugs received), the cost of further care at private outpatient facilities (public outpatient facilities were assumed to be free), and user fees associated with any inpatient care (which for simplicity we assumed was all public sector). The retail price (ie, cost to patients) of an RDT was calculated as US$0.33, and the retail prices of ACTs and antibiotics per course of treatment as US$1.02 and US$0.44, respectively. Non-medical patient costs (including travel costs) and indirect costs (including lost time and productivity) were not included due to lack of available data. Costs to retailers were not included, as we assumed that retailers would only participate if they could cover such costs from RDT and drug sales.

The calculation of supporting intervention costs is described in the online supplementary file S5. Community sensitisation, retailer training and retailer supervision costs were adapted from a similar intervention in 29 Ugandan drug shops.^18^ An additional waste collection cost was added, equal to the cost of supervision. The Uganda study reported an unusually low number of febrile patients, less than one febrile patient per outlet per day.^29^ In our base-case analysis, we increased this to five febrile patients per outlet per day and tested the impact of this assumption via sensitivity analysis. This reduces the number of outlets required to be trained and supervised for the modelled cohort of 100 000 febrile patients. Including an assumed management and overhead cost, supporting intervention costs are estimated at US$0.43 (US$0.21–US$0.64) per febrile patient (US$773 per outlet per year) (table 2).

### Cost-effectiveness

We calculated incremental cost-effectiveness ratios (ICERs) as total incremental costs divided by total DALYs averted, for the intervention compared with the control. DALYs were calculated as the sum of years of life lost and years of life with disability, applying a discount rate of 3%,^44^ African life expectancy (2006) from WHO life tables,^45^ and Global Burden of Disease disability weights.^46^

There is considerable debate about the selection of appropriate cost-effectiveness thresholds.^47 48^ We compared ICERs for each of our treatment scenarios against six country-specific thresholds calculated by Ochalek *et al*, which incorporate individual country estimates of health opportunity costs.^49^ The six countries all received private sector ACT subsidies as part of the Private Sector Co-payment Mechanism.^13^ Ochalek *et al* employ four different approaches for estimating the impact of changes in health expenditure on morbidity and mortality; we use the mean of the thresholds calculated from these four approaches, for each country. All thresholds were converted to 2017 US dollars,^50^ giving thresholds (per DALY averted) of: Madagascar US$84, Uganda US$115, Nigeria US$182, Tanzania US$283, Ghana US$521 and Kenya US$630.

### Sensitivity analysis

We explored the impact of individual parameter uncertainty on cost-effectiveness, in terms of both ICERs and net monetary benefit (NMB), using deterministic sensitivity analysis. NMB is expressed as the incremental value of health benefits in monetary terms (calculated by multiplying DALYs averted by the value of such benefits at the appropriate cost-effectiveness threshold), minus the incremental costs of the intervention.^51^

We also conducted probabilistic sensitivity analysis (PSA) to ascertain the combined impact of parameter uncertainty on incremental cost-effectiveness. Probability distributions were assigned to relevant parameters, including beta distributions for binomial probabilities, Dirichlet distributions for multinomial probabilities, and gamma distributions for costs.^51^ Using Monte Carlo simulation, 10 000 samples were drawn from the parameter distributions to generate incremental cost and incremental DALYs averted at both 5% and 50% PfPR.

### Patient and public involvement

Neither patients nor the public were involved in the study.

## Results

At 5% PfPR, the majority (85.8%) of the cohort of 100 000 febrile patients had a viral infection, which is unaffected by antimalarial or antibiotic drugs; 9.2% had a bacterial infection only, 4.7% had malaria only, and 0.3% had malaria and bacterial coinfection. At 50% PfPR, 48% of patients had a viral infection, 2% bacterial infection only, 47% malaria only and 3% coinfection (online supplementary file S2).

Table 3 shows the incremental costs, health outcomes and cost-effectiveness of subsidised RDT introduction for each of the treatment scenarios at 5% and 50% PfPR.

**Table 3:** Incremental costs, outcomes and cost-effectiveness of introducing subsidised malaria RDTs for 100 000 febrile patients in three private retail sector treatment scenarios (three small-scale studies in Nigeria (TS-N), Tanzania (TS-T) and Uganda (TS-U)), at 5% and 50% PfPR (2017 US$)

	5% PfPR			50% PfPR		
	TS-N	TS-T	TS-U	TS-N	TS-T	TS-U
**Incremental health service costs (US$)**						
RDT	7462	7462	7462	7462	7462	7462
Initial treatment	4700	−3058	−1186	6070	−649	−375
Further care	−71	−733	1537	−3338	−2813	2464
Supporting intervention	42 931	42 931	42 931	42 931	42 931	42 931
**Total incremental health service costs**	55 022	46 602	50 743	53 125	46 931	52 481
**Incremental health service costs per febrile patient**	**0.55**	**0.47**	**0.51**	**0.53**	**0.47**	**0.52**
**Incremental patient OOP costs (US$)**						
RDT	13 530	13 530	13 530	13 530	13 530	13 530
Initial treatment	2740	−7282	−5773	4648	−2377	−3837
Further care	−31	−228	467	−1770	−1438	1193
**Total incremental patient OOP costs**	16 239	6020	8224	16 409	9714	10 886
**Incremental patient OOP costs per febrile patient**	**0.16**	**0.06**	**0.08**	**0.16**	**0.10**	**0.11**
**Total incremental societal costs (US$**)	71 261	52 622	58 967	69 534	56 646	63 368
**Incremental societal costs per febrile patient**	**0.71**	**0.53**	**0.59**	**0.70**	**0.57**	**0.63**
**Incremental intermediate outcomes**						
Of patients with malaria*, number (% change) that get ACT	1773 (211%)	474 (25%)	123 (8%)	17 726 (211%)	4737 (25%)	1228 (8%)
Of patients without malaria†, number (% change) that get ACT	21 467 (126%)	−15 698 (−40%)	−6025 (−20%)	11 298 (126%)	−8262 (−40%)	−3171 (−20%)
Of patients with malaria*, number (% change) that get other antimalarial (not ACT)	−1,585 (−57%)	−127 (−9%)	−497 (−23%)	−15 848 (−57%)	−1266 (−9%)	−4969 (−23%)
Of patients without malaria†, number (% change) that get other antimalarial (not ACT)	−25 457 (−45%)	−6265 (−21%)	−14 345 (−34%)	−13 472 (−45%)	−3304 (−22%)	−7879 (−35%)
Of patients with malaria*, number (% change) that get any antimalarial	188 (5%)	347 (10%)	−374 (−10%)	1879 (5%)	3471 (10%)	−3741 (−10%)
Of patients without malaria†, number (% change) that get any antimalarial	−3990 (−5%)	−21 963 (−32%)	−20 370 (−28%)	−2 174 (−6%)	−11 566 (−32%)	−11 051 (−29%)
Of patients with bacterial infection, number (% change) that get antibiotic	−483 (−26%)	−84 (−6%)	−351 (−10%)	−287 (−30%)	−29 (−4%)	−228 (−12%)
Of patients without bacterial infection‡, number (% change) that get antibiotic	−4620 (−27%)	−788 (−6%)	−3376 (−10%)	−5338 (−29%)	−601 (−4%)	−4185 (−12%)
**Incremental final health outcomes—DALYS averted**						
*Plasmodium falciparum* malaria	631	488	−373	1276	988	−754
Bacterial	−523	−93	−378	−115	−19	−81
Viral	0	0	0	0	0	0
Coinfection	6	9	−12	56	79	−107
**All febrile illness**	**114**	**404**	−**763**	1217	1047	−**942**
**Cost-effectiveness (US$)**						
**Health service costs per DALY averted**	**482**	**115**	**Dominated**	**44**	**45**	**Dominated**
**Societal cost per DALY averted**	**624**	**130**	**Dominated**	**57**	**54**	**Dominated**

*Patients with malaria: patients with malaria only plus patients with coinfection.

†Patients without malaria: patients with a non-malarial febrile illness (NMFI) (bacterial or viral).

‡Patients without bacterial infection: patients with viral NMFI plus patients with malaria only.

ACT, artemisinin combination therapy; DALY, disability-adjusted life year; OOP, out-of-pocket; PfPR, *Plasmodium falciparum* positivity rate; RDT, rapid diagnostic test.

### Intermediate outcomes

The different parameters used for initial treatment, based on the three treatment scenarios, led to different intermediate outcomes in terms of the use of ACTs, other antimalarials, all antimalarials, and antibiotics. The introduction of RDTs increased ACT use for patients with malaria in all three treatment scenarios. TS-T and TS-U also resulted in better targeting of ACTs, with reductions in the proportion of people without parasitaemia that received an ACT. This was due to a reduction in the likelihood of receiving an ACT with a negative test compared with the control arm. In TS-N, the intervention more than doubled the number of people without malaria that received an ACT—largely due to the much higher likelihood that an untested patient would receive an ACT in the intervention arm (49%) compared with patients in the control arm (18%).

In all three treatment scenarios, RDT introduction led to modest reductions in antibiotic use in test-negative patients, including those with assumed bacterial infection. This is contrary to evidence from other public and private sector studies indicating that RDTs may lead to overall increases in antibiotic use by as much as 25 percentage points.^29 52 53^ We, therefore, conducted sensitivity analysis on the cost-effectiveness impact of substantially higher antibiotic use for test-negative patients (see Deterministic sensitivity analysis).

### Final health outcomes

In two treatment scenarios (TS-N and TS-T), the intervention led to a net reduction in deaths (online supplementary file S6). This was due to a reduction in deaths as a result of increased ACT or other antimalarial use in patients infected with malaria, and was more pronounced in the 50% PfPR setting. There was a comparatively modest increase in deaths relating to bacterial infection, primarily due to reduced antibiotic use. In TS-U, the intervention led to an increase in deaths from both malaria and bacterial infection. This was largely due to the reduction in the likelihood of receiving either an antimalarial or an antibiotic for the majority (59%) of patients in the intervention arm who were not tested. Consequently, the model predicted a wide range of estimates of DALYs averted across the treatment scenarios; at 50% PfPR, from 1217 DALYs averted for TS-N to 942 DALYs incurred for TS-U.

### Costs

Incremental health service costs were positive for all three treatment scenarios in both transmission settings (5% and 50% PfPR), largely as a result of supporting intervention and RDT subsidy costs in the intervention arm. Supporting intervention costs were by far the largest component, comprising 78%–92% of total health service costs. TS-N had the highest incremental health service cost in both the transmission settings shown (eg, US$55 022 at 5% PfPR) due to the cost of the ACT subsidy for the large increase in ACT use in the intervention arm.

Unlike the other treatment scenarios, TS-U had a positive incremental health service cost for further care (eg, US$1537 at 5% PfPR). This is due to fewer patients in the intervention arm receiving appropriate treatment and therefore progressing to severe disease, compared with the control arm.

Incremental patient out-of-pocket costs were driven primarily by RDT costs, based on a retail price of US$0.33 per test. Incremental patient out-of-pocket costs comprised 11%–24% of total incremental societal costs (combined health service costs and patient out-of-pocket costs).

### Cost-effectiveness

The base case ICER (health service perspective) at 5% PfPR was US$482 and US$115 per DALY averted for TS-N and TS-T, respectively. At 50% PfPR, the base case ICER was US$44 (TS-N) and US$45 (TS-T) per DALY averted. For TS-U, the intervention was dominated (ie, less effective and more expensive than the control) at both 5% and 50% PfPR (table 3). Comparing the ICERs against the six country-specific thresholds, both TS-N and TS-T would be considered cost-effective for all six countries at 50% PfPR. At 5% PfPR, TS-T would be considered cost-effective for all six countries except Madagascar, but TS-N would only be considered cost-effective for two countries (Kenya and Ghana). Results from a societal perspective follow a similar pattern but as one would expect are somewhat less cost-effective, reflecting the additional patient out-of-pocket medical costs included (table 3).

### Sensitivity analysis

#### Deterministic sensitivity analysis

NMB (from a health service perspective) of the three treatment scenarios across the range of PfPR (0%–90%) is shown in the online supplementary file S7. NMB has been calculated using a value of health benefit equal to the lowest and highest of the six country-specific cost-effectiveness threshold—Madagascar (US$84) and Kenya (US$630), respectively. In the two non-dominated treatment scenarios, TS-N and TS-T, the intervention is more cost-effective (ie, NMB increases) at higher levels of PfPR. This is primarily due to increased ACT use in malaria patients in the intervention arm compared with the control, the impact of which is more pronounced as PfPR (and therefore the proportion of patients with malaria) increases. NMB becomes positive (and the intervention cost-effective) at the point where the value of incremental health benefit generated exceeds incremental cost. In the medium/high transmission setting, NMB is positive for TS-T across the full range of PfPR at the Kenya threshold, and above 30% PfPR at the Madagascar threshold; NMB is positive for TS-N above 20% PfPR at the Kenya threshold and 35% PfPR at the Madagascar threshold.

Table 4 illustrates the sensitivity of the ICER (from a health service perspective) to changes in individual parameters, for each treatment scenario at 5% and 50% PfPR. Results are relatively robust to changes in individual parameter values at 50% PfPR, but much less so in the lower transmission setting. At 5% PfPR, the intervention for TS-N is dominated at either the low or high bound of the plausible range of uncertainty for 7 of 16 the parameters shown; TS-T remains cost-effective for the majority of the country thresholds across most of the 16 parameters tested. TS-N and TS-T are particularly sensitive at 5% PfPR to uncertainty in the impact of RDTs on treatment received (antimalarial with positive RDT result, antibiotic with negative RDT result), as well as the probability that malaria becomes severe without (effective) treatment, the probability that a severe patient receives inpatient care, and the case fatality rate of untreated (and, to a lesser extent, treated) malaria. Both TS-N and TS-T are also sensitive to changes in the discount rate and in the supporting intervention cost per febrile patient. TS-U remains dominated across the range of all parameters tested, except where we assumed that the initial treatment parameters for patients not receiving a test would be unchanged between the control and intervention arms (ie, RDT introduction would not influence the initial treatment received by untested patients in the intervention arm), and at 5% PfPR where the probability that a patient with a negative test result receives an antibiotic increases by 25 percentage points.

**Table 4:** Deterministic sensitivity of incremental health service cost per DALY averted to changes in 16 key model parameters (2017 US$)

Parameter	Best estimate (low-high)	Source for range (if different to PSA range)	5% PfPR						50% PfPR					
			TS-N		TS-T		TS-U		TS-N		TS-T		TS-U	
			Low	High	Low	High	Low	High	Low	High	Low	High	Low	High
Base case ICER	(Best estimates for each parameter)	(See table 2, online supplementary file S2)	482		115		D		44		45		D	
Discount rate	0.03 (0.01–0.10)	Assumptions	290	1372	73	309	D	D	27	120	28	122	D	D
Proportion of patients under 5 years	0.40 (0.30–0.50)	(See table 2)	636	389	128	105	D	D	51	38	53	39	D	D
Patients receiving RDT (‘uptake’)*	0.41 (0.08–0.72)	(See table 2)	1401	316	109	121	D	D	38	49	82	33	D	D
Positive RDT gets antimalarial	(See online supplementary file S4)		D	154	280	72	D	D	76	30	87	30	D	D
Antimalarial with positive RDT result that is ACT			709	362	128	105	D	D	47	41	49	42	D	D
Negative RDT gets antibiotic			D	47	233	31	D	196	45	37	47	37	D	D
Initial treatment parameters for intervention with no test unchanged from control			492		268		868		107		59		210	
Reduction in ACT effectiveness due to reduced API consumed	Low transmission: 0.15 (0.00–0.30) Med/high transmission: 0.10 (0.00–0.30)	Assumptions	103	D	90	159	D	D	30	129	39	57	D	D
Reduction in other antimalarial effectiveness due to reduced API consumed			D	131	123	109	D	D	59	31	47	42	D	D
Malaria case progresses to severe with no (or not effective) treatment*	(See PSA ranges in table 2 for <5 and >5 years, low and medium/high transmission settings)		D	40	701	34	D	D	129	5	111	6	D	D
Severe case receives further (inpatient) care*	0.75 (0.19–0.88)	(See table 2)	93	16 013	48	172	D	D	22	58	23	59	D	D
CFR of severe malaria receiving inpatient care *	0.10 (0.05–0.15)		5170	259	144	97	D	D	56	36	56	38	D	D
CFR of severe malaria with no further care *	(See PSA ranges in table 2 for <5 and >5 years, low and medium/high transmission settings)		D	216	236	91	D	D	87	29	81	31	D	D
RDT exmanufacturer price *	0.22 (0.17–0.28)	(See table 2)	471	495	112	119	D	D	43	45	44	46	D	D
ACT exmanufacturer price*	0.68 (0.51–1.56)		472	535	117	106	D	D	42	50	45	44	D	D
Supporting intervention cost per febrile patient	0.43 (0.21–2.54)	Min: −25%; Max: assumption based on^18^	294	2330	62	638	D	D	26	217	24	247	D	D
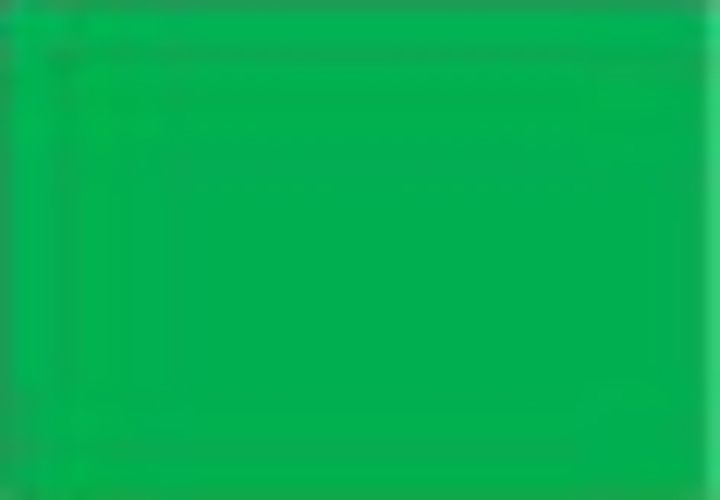	Cost-effective at all six country-specific thresholds (Madagascar, Uganda, Nigeria, Tanzania, Ghana and Kenya)													
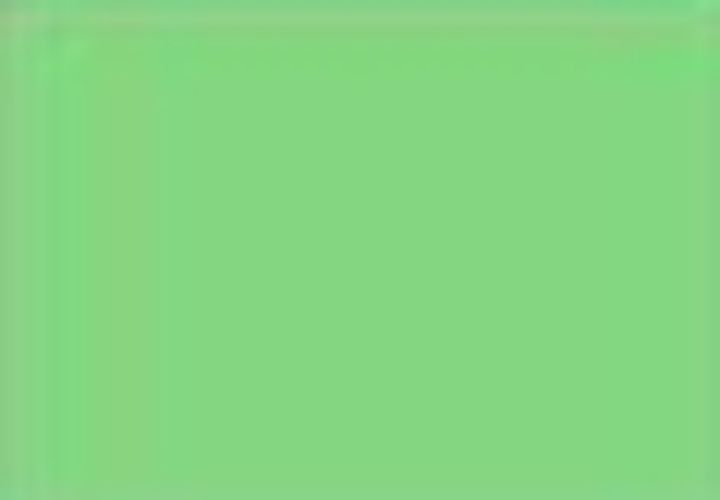	Cost-effective for at least one country-specific threshold (but not all six)													
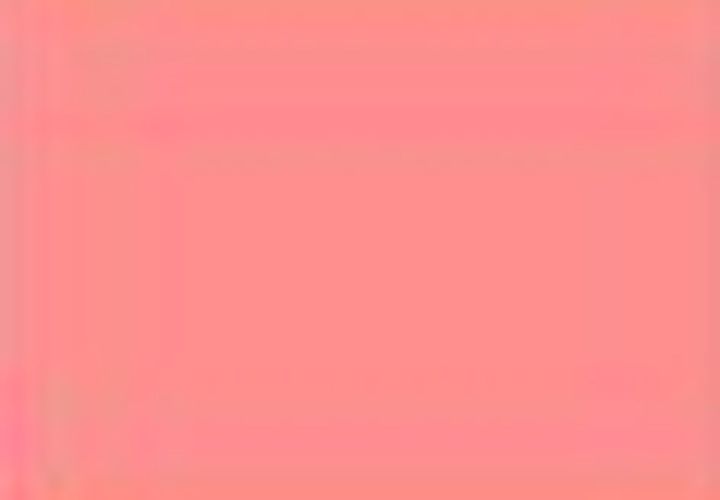	Not cost-effective at any of six country-specific thresholds, but not dominated													
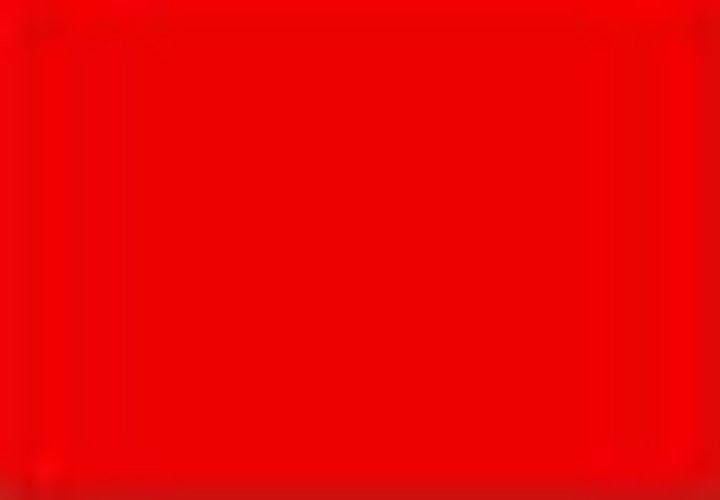	Dominated													

*Parameters where range of uncertainty in deterministic sensitivity analysis is the same as the PSA range. D: dominated, that is, intervention is more costly and less effective than the control.

ACT, artemisinin combination therapy; API, active pharmaceutical ingredient; CFR, case fatality rate; DALY, disability-adjusted life year; ICER, incremental cost-effectiveness ratio; PfPR, *Plasmodium falciparum* positivity rate; PSA, probabilistic sensitivity analysis; RDT, rapid diagnostic test.

#### Probabilistic sensitivity analysis

**Figure 3:**
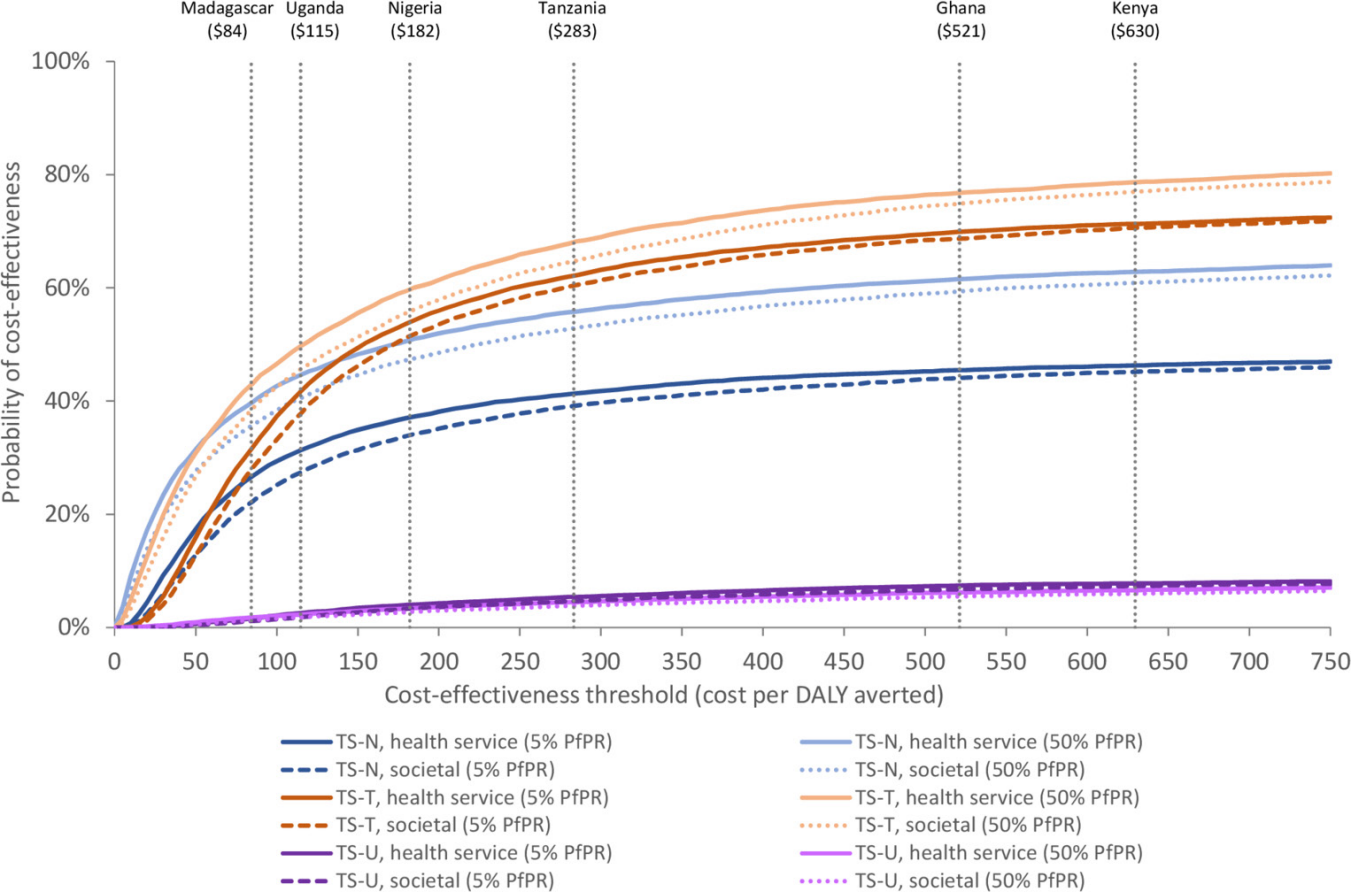
Cost-effectiveness acceptability curves; at 5% PfPR and 50% PfPR (2017 US$) Vertical dotted lines show country-specific cost-effectiveness thresholds for Madagascar (US$84), Uganda (US$115), Nigeria (US$182), Tanzania (US$283), Ghana (US$521) and Kenya (US$630). DALY, disability-adjusted life year; PfPR, *Plasmodium falciparum* positivity rate.

Individual results of the 10 000 simulations generated by the PSA are provided in the online supplementary file S8. Cost-effectiveness acceptability curves were calculated from 10 000 simulations generated by the PSA (figure 3). At 5% PfPR, the probability of cost-effectiveness at the highest threshold, Kenya (US$630), is 47% for TS-N and 72% for TS-T, from a health service perspective. The probability of cost-effectiveness is higher at 50% PfPR: 64% for TS-N and 80% for TS-T. TS-U never approaches a material probability of cost-effectiveness even at the highest of the six country thresholds. The probability of cost-effectiveness is reduced for all treatment scenarios when patient out-of-pocket costs are included.

## Discussion

In this paper, we have proposed a number of methodological developments in modelling malaria RDT introduction, and presented novel results on the cost-effectiveness of subsidised RDT introduction, in the context of an existing ACT subsidy in the SSA private sector. The results of our modelling were not conclusive on whether subsidised RDT introduction is cost-effective in such circumstances. The three small-scale studies we drew on for initial treatment parameters varied considerably in terms of how patients were treated in relation to whether or not they had a test and the test result, and this resulted in very different cost-effectiveness results for the three treatment scenarios. This is explored in more detail below. Moreover, implementation has yet to be conducted at a national scale, meaning that there is limited evidence on the feasibility, effectiveness and cost in operational settings,^8^ and it is possible that the growth in awareness and acceptance of RDTs over time may also influence these outcomes.

The three studies from which data were drawn for the treatment received (ie, the treatment scenarios) were all conducted in the context of substantial subsidisation of RDTs and ACTs, and with supporting intervention costs borne by the health service. Since these studies were conducted, ACT subsidies have been reduced in many countries.^13 54^ This may limit the generalisability of results, particularly in settings with a lower level of ACT subsidisation or none at all. Without an ACT subsidy, overall antimalarial use and the proportion of antimalarials that are ACTs may be lower, which may reduce the impact of RDT introduction on increased antimalarial (and particularly ACT) use. Without subsidisation of RDTs and supporting interventions, RDT uptake would likely be lower, reducing the impact of RDT introduction on initial treatment received by patients in the intervention arm. This underscores the need for further studies at scale of RDT use in the retail sector, and particularly in settings where private sector ACT subsidies are low or non-existent.

The differences in cost-effectiveness between treatment scenarios and transmission settings are underpinned by the substantial differences in the impact of RDTs on the treatment received in each scenario. In both TS-N and TS-T, RDT introduction leads to an overall increase in antimalarial use for malaria cases (+5% and +10% respectively), but the increase in ACT use for malaria cases is much higher for TS-N (+211%) than TS-T (+25%). For bacterial cases, antibiotic use is reduced by up to 30% in TS-N but as little as 4% in TS-T. As a result, improved health outcomes of malaria cases due to increased antimalarial use are higher for TS-N, and diminished health outcomes of bacterial cases due to reduced antibiotic use are less pronounced for TS-T than for TS-N. These differences help explain why both scenarios are more cost-effective in higher transmission settings, where the proportion of malaria cases is higher (and the proportion of bacterial cases is lower), and also why TS-N is more cost-effective than TS-T at 5% PfPR and vice versa at 50% PfPR.

Our analysis has a number of other limitations. As with all models of this kind, parameters are subject to a high amount of uncertainty. While the range of possible values for each parameter was assessed and incorporated into the PSA, the true confidence levels of many of these values are not known precisely. The probabilities of progression to severe illness and case fatality rates for untreated severe illness were taken from a Delphi study that reported substantial variation in estimates between experts.^23^ Disease progression for patients receiving ineffective treatment was assumed to be the same as for those where no treatment was received. Supporting intervention costs were adapted and scaled up from a single drug shop study in Uganda,^6^ adjusted based on an assumed number of febrile patients per outlet per day.

The model assumes that, in any patient who is parasitaemic, symptoms are causally attributable to either malaria alone or a malaria and bacterial coinfection. However, asymptomatic malaria parasitaemia is common in high transmission settings.^55^ In such cases, symptoms may be due to an acute self-resolving viral infection. For such patients, whether or not they received an antimalarial would make no difference to their clinical outcome. The model may, therefore, overstate the incremental effectiveness of the intervention in resolving symptoms of parasitaemic patients. Uncertainty relating to aetiology of fever is partly incorporated in the wide range of uncertainty modelled for antimalarial effectiveness and disease progression parameters, including a lower probability of disease progression in medium/high transmission than low transmission settings. However, the cost-effectiveness of RDT introduction may nevertheless be overstated for patients with asymptomatic malaria, and in settings where the proportion of such patients is high.

We assumed no change in initial treatment-seeking behaviour as a result of the intervention. It is possible that RDT introduction would encourage a greater proportion of febrile patients to seek treatment in the retail sector, with implications for further care-seeking and health outcomes.^56^ We also assumed that the true underlying diagnosis profile of untested patients in the intervention arm was the same as that for the population in the control arm. In practice, untested patients in the intervention arm may have less serious illnesses than the control arm, as they were not tested despite the availability of RDTs. The model also does not incorporate the potential impact of RDT introduction on antimalarial and antibiotic resistance, or any possible side effects of treatment. We also do not include the potential benefits of retail sector RDT introduction for enhancing sector-wide malaria surveillance, were such data to be integrated into existing systems.

Our findings challenge the traditional rationale of RDTs as primarily to reduce inappropriate antimalarial use and improve case management of NMFIs.^29 57–59^ This view was informed by public sector models indicating that RDTs are more cost-effective in lower transmission settings where there are relatively few malaria cases and antimalarials are overused and untargeted.^20^ However, our analysis shows that where RDTs lead to an increase in ACT (or other antimalarial) use, particularly for patients with a positive RDT result, improved health outcomes for malaria cases can be a strong driver of cost-effectiveness. This is perhaps more likely in the retail sector, where pre-RDT antimalarial use is relatively low compared with the public health sector.^60^

TS-U is dominated across the range of PfPR, as the intervention results in an 11% reduction in antimalarials for malaria cases (despite an 8% increase in ACTs for this group) and a 10% reduction in antibiotics for bacterial cases. This largely relates to initial treatment received by the untested group in the intervention arm, where the use of other (non-ACT) antimalarials is a third lower, and antibiotic use 16% lower, than the control arm; TS-U was reasonably cost-effective at 50% PfPR when we assumed in the sensitivity analysis that the untested group in the intervention received the same treatment as the control. Based on our assumed uptake, almost three-fifths (59%) of patients in the intervention arm did not receive a test. TS-U shows the importance of this untested group in determining cost-effectiveness, and in strategies to promote RDT uptake. It also highlights the need to better understand what illnesses untested patients are likely to have, what treatment they receive, and their health outcomes.

This analysis also provides an insight into the importance of monitoring other (non-ACT) antimalarial use. Even though the efficacy of ACTs is considerably higher than for other antimalarials, non-ACT antimalarials are still commonly used in the retail sector in many settings. For a patient with malaria, receiving a non-ACT antimalarial may be more effective than not receiving any antimalarial at all. All treatment scenarios reported relatively high levels of other antimalarial use; in TS-U in particular, the reduction in non-ACT antimalarial use for untested patients in the intervention arm was a contributor to the intervention being dominated. Changes in other antimalarial use can have a material impact on cost-effectiveness, particularly in the retail sector where other antimalarials may comprise a larger proportion of total antimalarial use than in other settings. Therefore, it is important that other, non-ACT antimalarials continue to be monitored in future studies, particularly for large-scale implementations.

In the three treatment scenarios modelled, RDT introduction did not appear to have a strong impact on antibiotic use for RDT-negative patients. In all treatment scenarios and both transmission settings there was a modest reduction in antibiotic use in patients with a bacterial illness, compared with the control; this contributed to poorer health outcomes for bacterial cases and had a negative impact on cost-effectiveness. This reduced antibiotic use is in contrast to previous research showing a general increase in antibiotic prescribing in public and private intervention settings, particularly among patients with negative malaria tests^52^ and counter to expectations that RDTs would lead to improved management of NMFI cases. The absence of increased antibiotic use could be related to possible restrictions on antibiotic prescribing in the retail sector and the referral to other settings of patients testing negative. The reduction could also result from increased monitoring, and hence improved compliance, by retail outlets in the intervention arms. Given these results, we examined the impact of an increase of 25 percentage points in antibiotics received by patients with a negative test result—similar to the increase seen for test-negative patients in another retail sector study.^29^ Such an increase would improve the health outcomes of bacterial cases and substantially enhance the cost-effectiveness of RDT introduction in lower transmission settings. Nevertheless, it highlights the need for improved diagnosis of patients with bacterial infection in order to better target antibiotics to patients who need them.

## Conclusion

The cost-effectiveness of subsidised RDTs in the SSA retail sector is strongly influenced by treatment practices and how these are affected by RDT introduction, for which further evidence is required from larger-scale operational settings. Notwithstanding this, initial evidence suggests that the introduction of subsidised RDTs could promote the increased use of ACTs and other antimalarials in patients with malaria. As a result, RDTs may be cost-effective particularly in higher transmission settings, where a greater proportion of febrile patients have malaria and therefore benefit from increased antimalarial use.
