# Supplementary material for: Modelling the cost-effectiveness of introducing subsidised malaria rapid diagnostic tests in the private retail sector in sub-Saharan Africa
Source: BMJ Glob Health. 2020 May 20;5(5):e002138. doi: 10.1136/bmjgh-2019-002138 (PMC7247415; doi:10.1136/bmjgh-2019-002138)
Supplement: Supplementary data [file bmjgh-2019-002138supp001.pdf]

SUPPLEMENTARY FILE

S1. Decision tree model structure for patient with an uncomplicated febrile illness without obvious cause; (a) diagnosis, test result, and initial treatment, and (b) treatment effectiveness, disease progression and further care, and final health outcome *

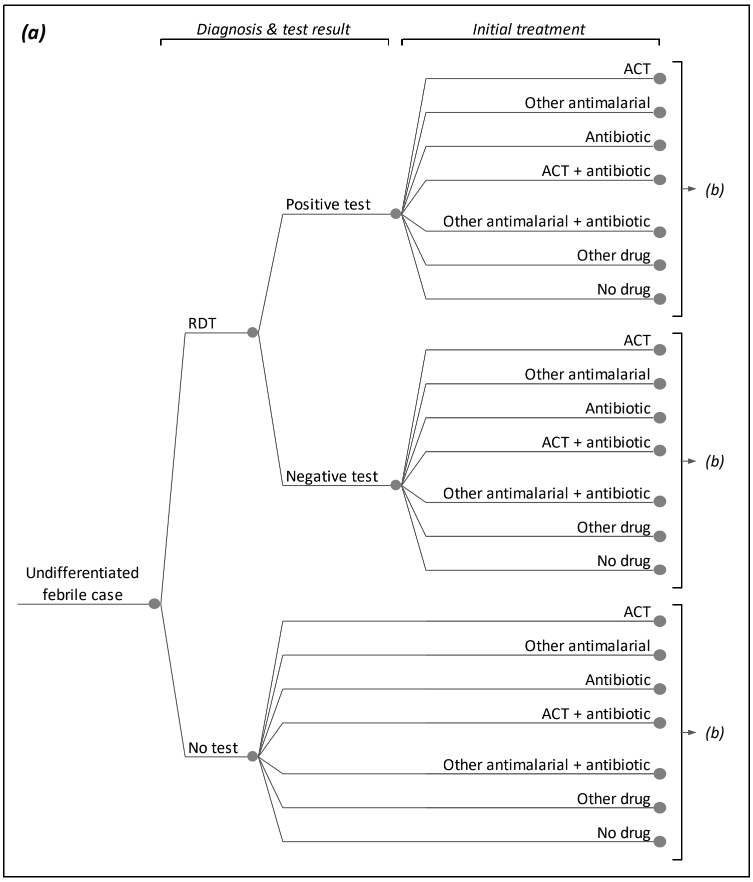

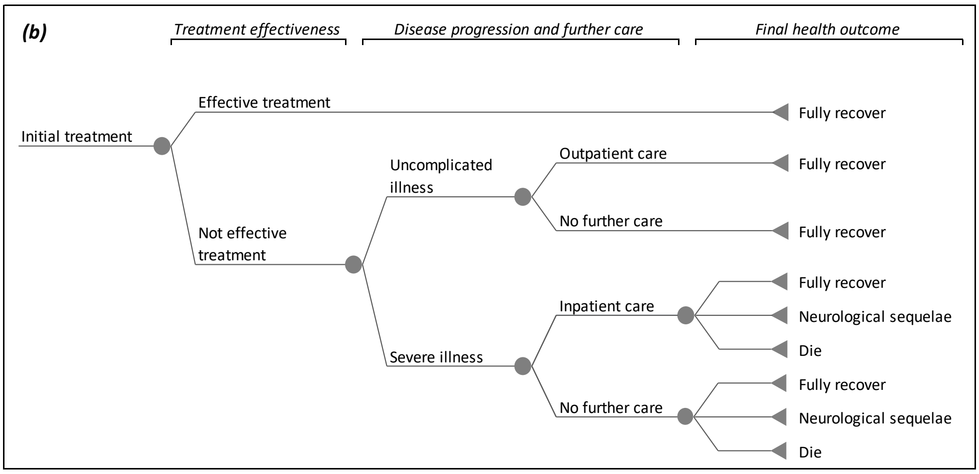

* Replicated for each of the four true underlying diagnoses modelled (malaria, bacterial infection, malaria and bacterial co-infection, and viral).

S-1

S2. Other cost-effectiveness model parameters

Parameter	Value (Range for PSA)	Distribution for PSA	Source(s)
Inflation rate	6.8%	Point estimate	Median of 6 country (Ghana, Kenya, Madagascar, Nigeria, Tanzania, Uganda) GDP deflator (5 year average, 2012-2017)(69)
Discount rate	3.0%	Point estimate	(44)
ACT efficacy (for malaria and bacterial co-infection)	0.05 (0.00-0.10)	Beta	Assumption
Other antimalarial efficacy (for malaria and bacterial co-infection)	0.05 (0.00-0.10)	Beta	Assumption
ACT + antibiotic efficacy (for malaria and bacterial co-infection)	0.85 (0.765-0.935)	Beta	Assumption
Other antimalarial efficacy (for malaria and bacterial co-infection)	0.60 (0.15-0.80)	Beta	Assumption
Antibiotic efficacy (for malaria and bacterial co-infection)	0.05 (0.00-0.10)	Beta	Assumption
Proportion of bacterial cases in low HIV setting	0.50 (0.00-1.00)	Uniform	Assumption
Malaria and bacterial co-infection case progresses to severe with no (or not effective) treatment<br><5 years<br>5+ years	Low HIV: / High HIV:<br>0.20 (0.05-0.80) / 0.40 (0.15-0.90)<br>0.20 (0.05-0.70) / 0.30 (0.10-0.90)	 Beta<br>Beta	Assumption: same as for bacterial infection
Uncomplicated patient with no (or not effective) treatment receives further (outpatient) care	0.48 (0.36-0.73)	Beta	(20)
Outpatient care provided in health centre (rather than hospital) setting	0.68 (0.51-0.93)	Beta	(20)
Probability of neurological sequelae with severe malaria<br><5 years<br>5+ years	 0.035 (0.01-0.05)<br>0.015 (0.01-0.02)	 Beta<br>Beta	(20)
Probability of neurological sequelae with severe bacterial infection, or malaria and bacterial co-infection<br><5 years<br>5+ years	 0.02 (0.016-0.024)<br>0.038 (0.01-0.07)	 Beta<br>Beta	(20)
CFR of severe malaria and bacterial co-infection receiving inpatient care	0.10 (0.05-0.15)	Beta	Assumption: same as for severe malaria receiving inpatient care

S-2

Parameter	Value (Range for PSA)		Distribution for PSA	Source(s)
CFR of severe malaria and bacterial co-infection with no further care	Low transmission:	Medium/high transmission:		Assumption: same as for severe malaria with no further care
<5 years	0.73 (0.25-0.95)	0.45 (0.05-0.90)	Beta	
5+ years	0.70 (0.30-0.95)	0.60 (0.10-0.90)	Beta	
RDT freight and insurance (% ex-manufacturer price)	10%		Point estimate	Assumption
RDT consumables cost (USD)	0.05 (0.04-0.06)		Beta	Assumption
RDT mark-up (% ex-manufacturer price)	200% (150-250%)		Uniform	Assumption
Child drug cost per dose (% adult cost per dose)	0.50 (0.375-0.625)		Beta	(20)
ACT mark-up (% ex-manufacturer price)	840% (630-1,050%)		Gamma	(14) (+/- 25%)
Other antimalarial ex-manufacturer price (USD)	0.34 (0.25-0.42)		Gamma	(67) (+/- 25%)
Antibiotic (amoxicillin) ex-manufacturer price (USD)	0.27 (0.25-0.34)		Gamma	IMPPG<br>(+/- 25%)
Other drug ex-manufacturer price (USD)	0.10 (0.05-0.13)		Gamma	Assumption
Antibiotic and other drug mark-up (% ex-manufacturer price)	100% (50-100%)		Uniform	Assumption
Artesunate (inpatient treatment) cost per adult dose, patient recovers (USD)	7.25 (5.44-9.06)		Gamma	(67)
Proportion of artesunate dose received where patient dies	0.65 (0.55-0.75)		Beta	Based on (20)
Consumables per artesunate dose (USD)	0.60 (0.45-0.75)		Gamma	Assumption (+/- 25%)
Microscopy diagnoses per inpatient visit	2		Point estimate	Assumption
Average length of inpatient stay (days), patient recovers	3.0 (2.0-5.0)		Gamma	(42)
Average length of inpatient stay (days), patient dies	2.0 (1.5-2.5)		Gamma	(20,43)
User fees paid by patient (% total inpatient cost)	0.20 (0.00-0.40)		Beta	Assumption
Outpatient cost per visit, hospital (excluding treatment costs) (USD)	1.55 (1.35-4.29)		Gamma	Median of 6 country (Ghana, Kenya, Madagascar, Nigeria, Tanzania, Uganda) outpatient visit cost, primary hospital(68)
Outpatient cost per visit, health centre * (USD)	1.13 (0.96-3.05)		Gamma	Median of 6 country (Ghana, Kenya, Madagascar, Nigeria, Tanzania, Uganda) outpatient visit cost, health centre (no beds)(68)
Microscopy cost, per outpatient presentation (USD)	0.84 (0.44-1.92)		Gamma	(20) (+/- 50%)
Proportion of outpatients presenting to public facility	0.40 (0.20-0.60)		Beta	Assumption

Parameter	Value (Range for PSA)	Distribution for PSA	Source(s)
Life expectancy of patient < 5 years (years)	63.55 (57.20-69.91)	Gamma	(45) (Expectation of life at age 1-4 years, based on previous models(20); mean of male and female values)
Life expectancy of patient > 5 years (years)	43.80 (39.42-49.18)	Gamma	(45) (Expectation of life at age 25-29 years, based on previous models(20); mean of male and female values)
Average duration of uncomplicated fever (days)	3 (1-7)	Gamma	Assumption
Average duration of severe fever (days)	14 (7-28)	Gamma	Assumption
Disability weight for uncomplicated fever	0.051 (0.032-0.074)	Gamma	(46) ('Infectious disease acute episode, moderate')
Disability weight for severe fever	0.542 (0.374-0.702)	Gamma	(46) ('Infectious disease acute episode, severe')
Disability weight for neurological sequelae	0.133 (0.088-0.190)	Gamma	(46) ('Motor plus cognitive impairments: severe')

CFR: case fatality rate. IMPPG: International Medical Products Price Guide (available from www.mshpriceguide.org).
* Outpatient cost is bed-day cost only; excludes cost of treatment. All costs were adjusted to 2017 US dollars using the median of the five year annual average GDP deflator in the six countries participating in the Private Sector Co-payment Mechanism(69).

S3. True underlying diagnosis of fever of population (100,000 febrile patients) at 5% and 50% PfPR

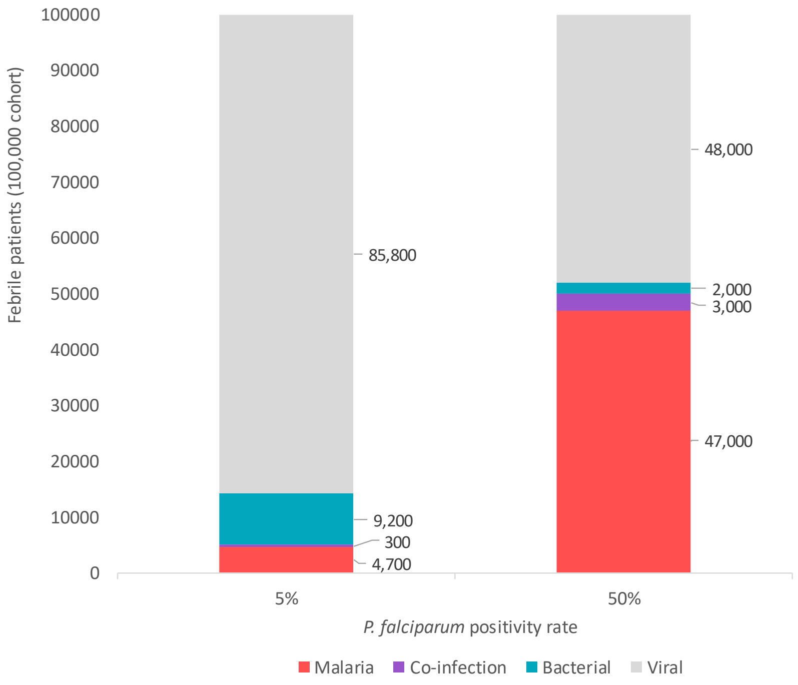

S4. Initial treatment received by mutually-exclusive treatment categories used in decision-tree model, for three treatment scenarios (TS-N, TS-T, TS-U); base case and four initial treatment sensitivities used for deterministic sensitivity analysis

Table 3 shows the results of deterministic sensitivity analysis for 15 parameters, including four initial treatment parameters:

- *Positive RDT result gets antimalarial:* varies (by +/- 10 percentage points) the probability that a patient with a positive RDT receives an antimalarial;
- *Antimalarial with positive RDT result that is ACT:* varies (by +/- 10 percentage points) the probability that a patient with a positive RDT and receiving an antimalarial receives an ACT (rather than a non-ACT antimalarial);
- *Negative RDT result gets antibiotic:* varies (by +25/-5 percentage points) the probability that a patient with a negative RDT receives an antibiotic; and
- *Initial treatment parameters for intervention with no test unchanged from control:* varies the probability of receiving a particular treatment for a patient not receiving a test in the intervention arm to match the probabilities of initial treatment for patients in the control arm (i.e. assumes that the introduction of RDTs would not influence the initial treatment received by untested patients in the intervention arm).

The tables below show the probabilities of receiving a particular initial treatment, at base case and for each of the above sensitivities. Initial treatment probabilities are shown as (i) the seven mutually-exclusive treatment categories used in the decision-model (ACT, other antimalarial, ACT + antibiotic, other antimalarial + antibiotic, antibiotic only, other drug, no drug), and (ii) 'summary' categories of the total probabilities of receiving an ACT, other antimalarial, all antimalarial, and antibiotic for the control arm and each test result in the intervention arm. Tables (a), (b), and (c) provide the values for TS-N, TS-T, and TS-U, respectively.

(a) **TS-N**(31,37)

	Sensitivity: Low/High	Categories in decision-tree								Summary categories			
		**ACT**	**Other AM**	**ACT+AB**	**Other AM + AB**	**AB**	**Other**	**None**	**Total**	**ACT**	**Other AM**	**All AM**	**AB**
**Base case**													
Control, no test		15%	51%	3%	9%	7%	10%	5%	100%	18%	60%	77%	19%
Intervention, no test		41%	31%	8%	4%	1%	6%	9%	100%	49%	35%	84%	13%
Intervention, positive test		56%	11%	12%	1%	1%	1%	19%	100%	68%	11%	79%	13%
Intervention, negative test		23%	27%	5%	2%	9%	15%	19%	100%	27%	29%	56%	16%
**Sensitivity: Positive RDT results gets antimalarial (+/- 10%)**													
Intervention, positive test	Low (-10%)	49%	9%	10%	0%	2%	2%	27%	100%	59%	10%	**69%**	13%
Intervention, positive test	High (+10%)	63%	12%	14%	1%	0%	1%	10%	100%	77%	13%	**89%**	13%
**Sensitivity: Antimalarial with positive RDT result that is ACT**													
Intervention, positive test	Low (-10%)	49%	18%	11%	1%	2%	1%	18%	100%	**60%**	**19%**	79%	13%
Intervention, positive test	High (+10%)	63%	3%	13%	0%	0%	1%	20%	100%	**76%**	**3%**	79%	13%
**Sensitivity: Negative RDT result gets antibiotic**													
Intervention, negative test	Low (-5%)	23%	27%	5%	2%	4%	17%	22%	100%	27%	29%	56%	**11%**
Intervention, negative test	High (+25%)	23%	27%	5%	2%	34%	4%	5%	100%	27%	29%	56%	**41%**
**Sensitivity: Initial treatment parameters for intervention with no test unchanged from control**													
Intervention, no test		15%	51%	3%	9%	7%	10%	5%	100%	**18%**	**60%**	**77%**	**19%**

(b) TS-T(30,38)

	Sensitivity: Low/High	Categories in decision-tree								Summary categories			
		**ACT**	**Other AM**	**ACT+AB**	**Other AM + AB**	**AB**	**Other**	**None**	**Total**	**ACT**	**Other AM**	**All AM**	**AB**
**Base case**													
Control, no test		34%	28%	7%	3%	6%	19%	4%	100%	41%	31%	72%	15%
Intervention, no test		28%	33%	9%	4%	4%	17%	4%	100%	37%	38%	75%	17%
Intervention, positive test		67%	13%	8%	1%	1%	2%	7%	100%	75%	14%	90%	10%
Intervention, negative test		3%	4%	0%	0%	9%	42%	42%	100%	3%	4%	7%	9%
**Sensitivity: Positive RDT results gets antimalarial (+/- 10%)**													
Intervention, positive test	Low (-10%)	60%	12%	7%	1%	2%	4%	14%	100%	67%	13%	**80%**	10%
Intervention, positive test	High (+10%)	74%	15%	9%	1%	0%	0%	0%	100%	84%	16%	**100%**	10%
**Sensitivity: Antimalarial with positive RDT result that is ACT**													
Intervention, positive test	Low (-10%)	59%	22%	7%	2%	1%	2%	7%	100%	**66%**	**23%**	90%	10%
Intervention, positive test	High (+10%)	75%	5%	9%	0%	1%	2%	7%	100%	**84%**	**5%**	90%	10%
**Sensitivity: Negative RDT result gets antibiotic**													
Intervention, negative test	Low (-5%)	3%	4%	0%	0%	4%	45%	44%	100%	3%	4%	7%	**4%**
Intervention, negative test	High (+25%)	3%	4%	0%	0%	34%	30%	29%	100%	3%	4%	7%	**34%**
**Sensitivity: Initial treatment parameters for intervention with no test unchanged from control**													
Intervention, no test		34%	28%	7%	3%	6%	19%	4%	100%	**41%**	**31%**	**72%**	**15%**

Bath D, *et al. BMJ Global Health* 2020; 5:e002138. doi: 10.1136/bmjgh-2019-002138

(c) TS-U(28,36)

	Sensitivity: Low/High	Categories in decision-tree								Summary categories			
		**ACT**	**Other AM**	**ACT+AB**	**Other AM + AB**	**AB**	**Other**	**None**	**Total**	**ACT**	**Other AM**	**All AM**	**AB**
**Base case**													
Control, no test		25%	26%	8%	19%	10%	13%	0%	100%	32%	45%	77%	37%
Intervention, no test		24%	19%	8%	11%	13%	26%	0%	100%	32%	30%	61%	31%
Intervention, positive test		30%	25%	11%	18%	3%	9%	4%	100%	41%	42%	83%	31%
Intervention, negative test		15%	13%	2%	15%	19%	23%	15%	100%	17%	27%	44%	35%
**Sensitivity: Positive RDT results gets antimalarial (+/- 10%)**													
Intervention, positive test	Low (-10%)	27%	22%	10%	15%	6%	14%	6%	100%	36%	37%	**73%**	31%
Intervention, positive test	High (+10%)	34%	28%	12%	20%	0%	5%	2%	100%	46%	47%	**93%**	31%
**Sensitivity: Antimalarial with positive RDT result that is ACT**													
Intervention, positive test	Low (-10%)	24%	30%	9%	21%	2%	10%	5%	100%	**33%**	**51%**	83%	31%
Intervention, positive test	High (+10%)	36%	20%	13%	14%	4%	9%	4%	100%	**49%**	**34%**	83%	31%
**Sensitivity: Negative RDT result gets antibiotic**													
Intervention, negative test	Low (-5%)	15%	13%	2%	15%	14%	26%	17%	100%	17%	27%	44%	**30%**
Intervention, negative test	High (+25%)	15%	13%	2%	15%	44%	8%	5%	100%	17%	27%	44%	**60%**
**Sensitivity: Initial treatment parameters for intervention with no test unchanged from control**													
Intervention, no test		25%	26%	8%	19%	10%	13%	0%	100%	**32%**	**45%**	**77%**	**37%**

S5. Supporting intervention costs: parameters of introducing subsidised malaria RDTs in the private retail sector (2017 USD)*

Parameter	Value	Source(s)
Community sensitisation, per patient (USD)	0.17	(18)
Retailer training, per patient (USD)	0.85	(18)
Retailer supervision, per patient (USD)	0.70	(18)
Waste collection, per patient (USD)	0.70	Assumption
**Total supporting intervention costs, per patient (USD)**	**2.42**	
Number of patients per day	0.8	(29)
Adjusted number of patients per day	5.0	Assumption
Overhead (%)	5%	Assumption
**Adjusted supporting intervention costs, per patient, including overhead (USD)**	**0.43 (0.21-0.64)**	(Range +/- 50%)

* All costs were adjusted to 2017 US dollars using the median of the five year annual average GDP deflator in the six countries participating in the Private Sector Co-payment Mechanism(69).

Bath D, *et al. BMJ Global Health* 2020; 5:e002138. doi: 10.1136/bmjgh-2019-002138

S6. Incremental deaths averted for 100,000 febrile patients in three private retail sector treatment scenarios (TS-N, TS-T, TS-U), at 5% and 50% PfPR

	**5% PfPR**			**50% PfPR**		
	**TS-N**	**TS-T**	**TS-U**	**TS-N**	**TS-T**	**TS-U**
Incremental final health outcomes – deaths averted						
P. falciparum malaria	23	17	-13	43	33	-26
Bacterial	-19	-3	-13	-4	-1	-3
Viral	-	-	-	-	-	-
Co-infection	-	-	-	2	3	-4
**All febrile illness**	**4**	**14**	**-27**	**41**	**36**	**-32**

S7. Deterministic sensitivity of net monetary benefit, health service perspective, at cost-effectiveness thresholds for Madagascar ($84) and Kenya ($630), to changes in *P. falciparum* positivity rate (0-90%), across three treatment scenarios (TS-N, TS-T, TS-U) (2017 USD)

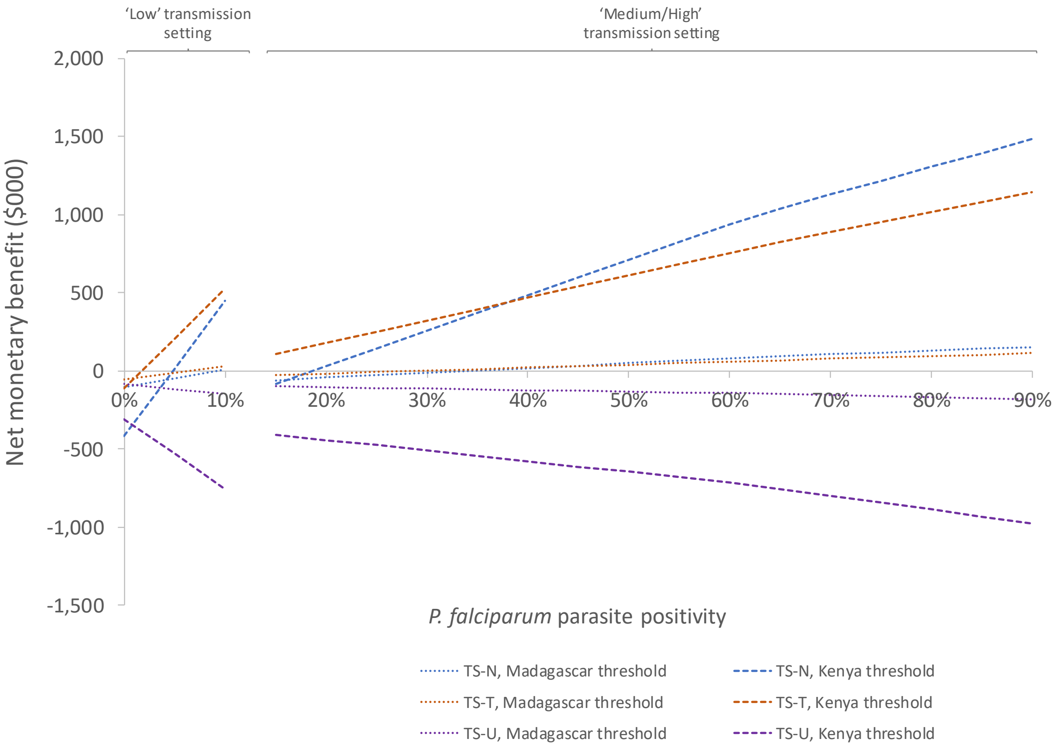

The light-dash lines show net monetary benefit (NMB) for each of the three treatment scenarios, calculated using a cost-effectiveness threshold for Madagascar ($84), across a range of the P. falciparum positivity rate (PfPR). The heavy-dash lines show NMB for the treatment scenarios using a a cost-effectiveness threshold for Kenya ($630).

S8. Probabilistic sensitivity (10,000 simulations) of incremental health service costs and incremental DALYs averted; at (a) 5% PfPR and (b) 50% PfPR (2017 USD)

(a)

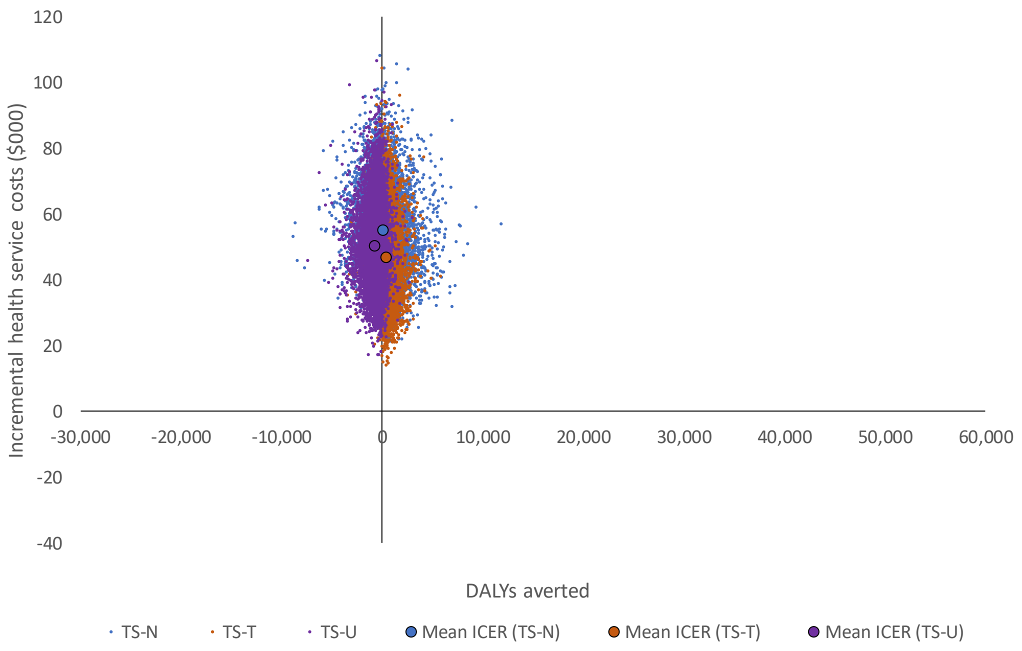

(b)

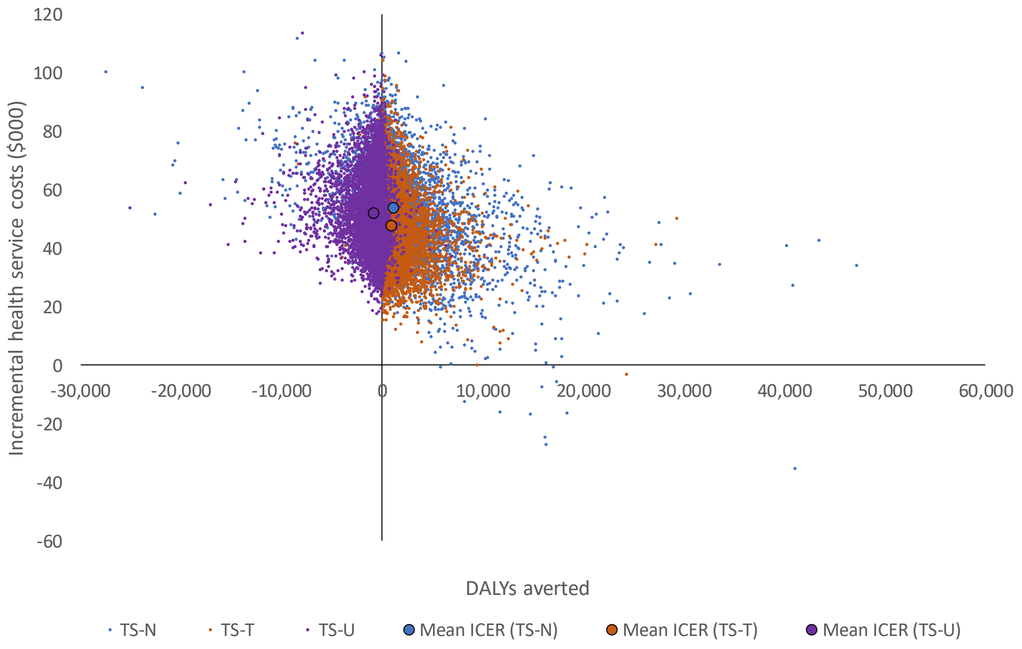

Each small dot represents one of the 10,000 simulations, for each of the three treatment scenarios, in each transmission setting. The large dots show the mean ICER for each treatment scenario.
